# Supplementary material for: Comparative characterization of Cas12f orthologs reveals mechanistic features underlying enhanced genome editing efficiency
Source: Nat Struct Mol Biol. 2026 Apr 13;33(5):756–67. doi: 10.1038/s41594-026-01788-6 (PMC13186708; doi:10.1038/s41594-026-01788-6)

nature structural & molecular biology

Supplementary information https://doi.org/10.1038/s41594-026-01788-6

Comparative characterization of Cas12f orthologs reveals mechanistic features underlying enhanced genome editing efficiency

In the format provided by the authors and unedited

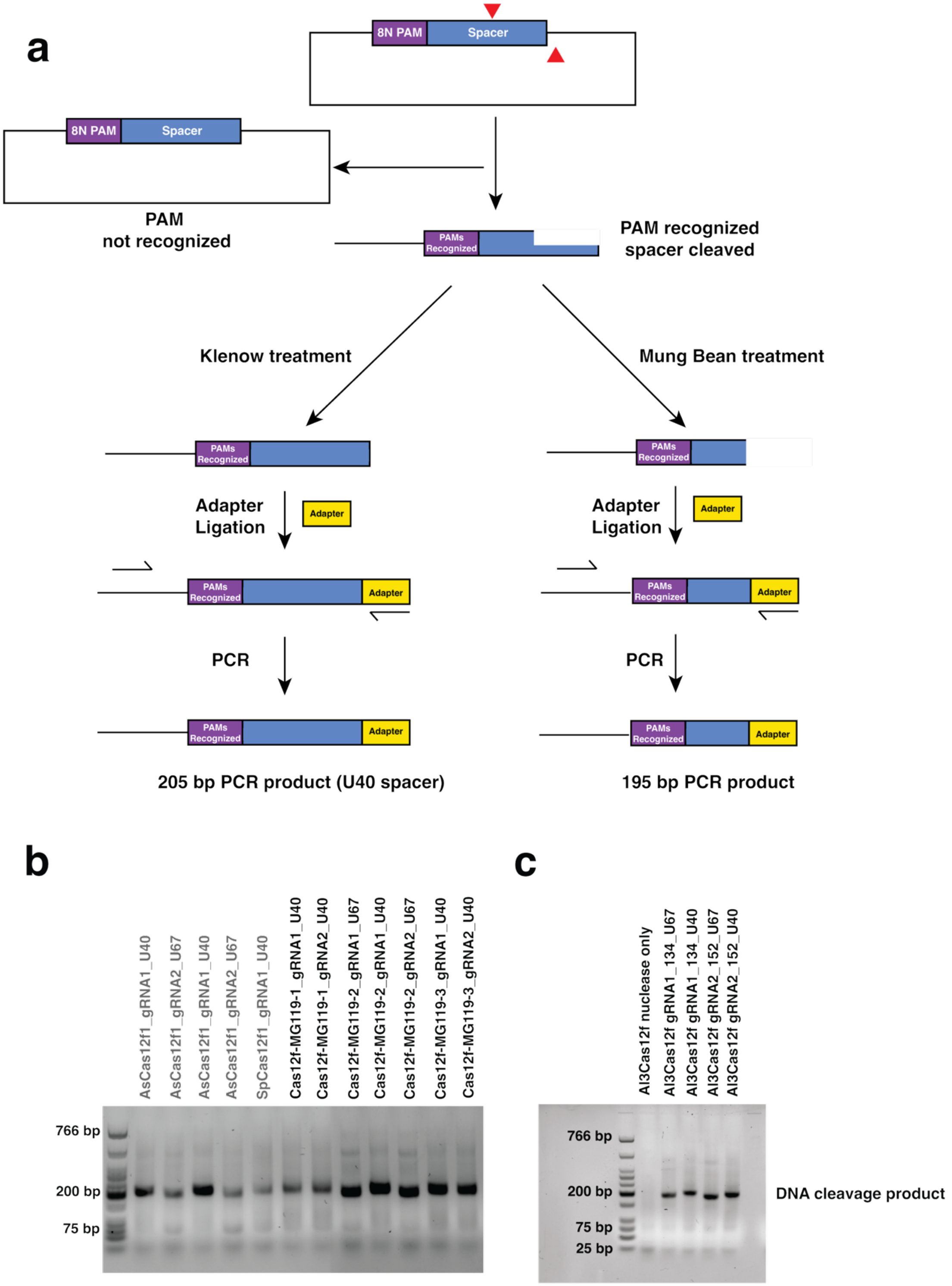

a
8N PAM
Spacer
8N PAM
Spacer
PAM
not recognized
PAMs Recognized
PAM recognized
spacer cleaved
Klenow treatment
Mung Bean treatment
PAMs Recognized
PAMs Recognized
Adapter
Ligation
Adapter
Adapter
Ligation
Adapter
PAMs Recognized
Adapter
PAMs Recognized
Adapter
PCR
PCR
PAMs Recognized
Adapter
PAMs Recognized
Adapter
205 bp PCR product (U40 spacer)
195 bp PCR product
b
AsCas12f1_gRNA1_U40
AsCas12f1_gRNA2_U67
AsCas12f1_gRNA1_U40
AsCas12f1_gRNA2_U67
SpCas12f1_gRNA1_U40
Cas12f-MG119-1_gRNA1_U40
Cas12f-MG119-1_gRNA2_U40
Cas12f-MG119-2_gRNA1_U67
Cas12f-MG119-2_gRNA1_U40
Cas12f-MG119-2_gRNA2_U67
Cas12f-MG119-3_gRNA1_U40
Cas12f-MG119-3_gRNA2_U40
766 bp
200 bp
75 bp
c
Al3Cas12f nuclease only
Al3Cas12f gRNA1_134_U67
Al3Cas12f gRNA1_134_U40
Al3Cas12f gRNA2_152_U67
Al3Cas12f gRNA2_152_U40
766 bp
200 bp
75 bp
25 bp
DNA cleavage product

Supplementary Figure 1. Validation of the gRNA designs.

a. Schematic of experimental design. **b**. *In vitro* cleavage of an 8N PAM plasmid library with MG nucleases and publicly available Cas12f orthologs using gRNAs designs of different lengths (i.e., gRNA1 vs gRNA2) and spacers with two different GC contents (i.e., U40 and U67), transcribed and translated with PURExpress. Activity was confirmed in 2% agarose gels (NEB Low Molecular Weight DNA Ladder) with cleavage products at 188 bp (U67 spacer) or 205 bp (U40 spacer). The cleaved products were also sequenced by NGS to confirm the PAM sequence. **c.** Predicted Al3Cas12f gRNA designs were tested for two different gRNA lengths (i.e.,134 nt and 152 nt) with the same two spacers targeting an 8N PAM library (See Methods section for further details). Uncropped gel images are provided in Source Data section at the end of this file.

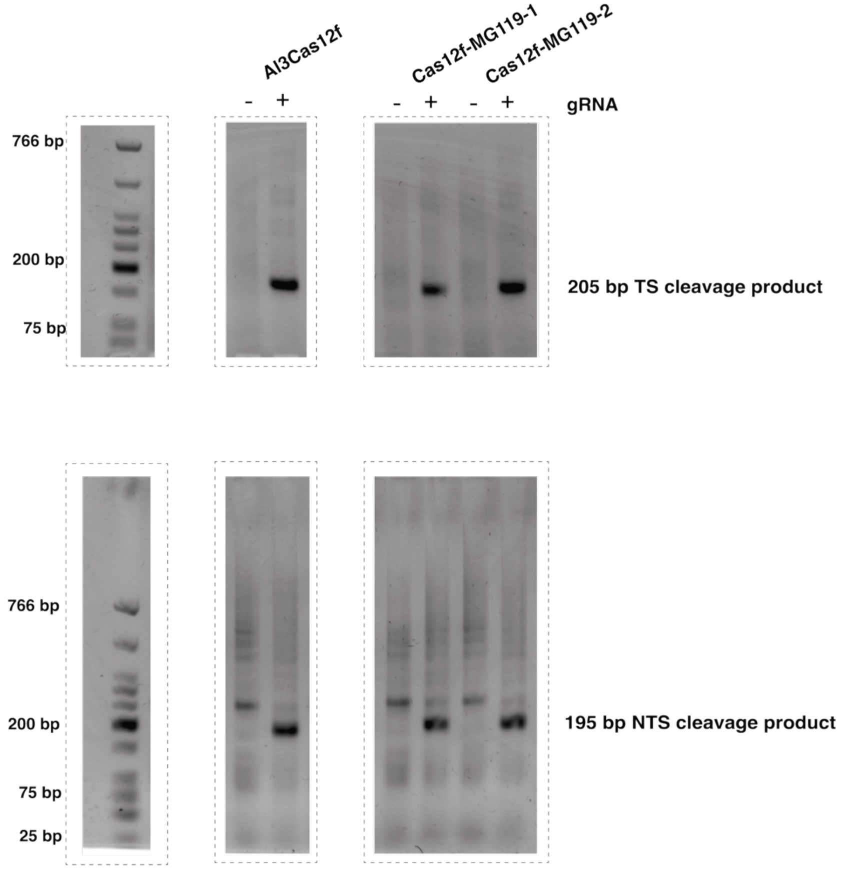

Supplementary Figure 2. Double strand cleavage products.

In vitro cleavage of an 8N PAM library with active MG gRNAs designs (134 nt Al3Cas12f gRNA,145 nt Cas12f MG119-1 gRNA, 129 nt Cas12f MG119-2 gRNA) and U40 spacer, transcribed and translated with PURExpress. Activity was confirmed in 2% agarose gels (NEB Low Molecular Weight DNA Ladder). Target strand (TS) cleavage sites were captured by 205 bp products generated with the DNA polymerase I large Klenow fragment (top). Non target strand (NTS) cleavage sites were captured by 195 bp products generated with Mung Bean nuclease (bottom; see Methods section for further details). Left lane (-): nuclease only. Right lane (+): nuclease plus gRNA. The cut sites were confirmed by NGS, as shown in **Extended Data Fig. 1.** Uncropped gel images are provided in Source Data section at the end of this file.

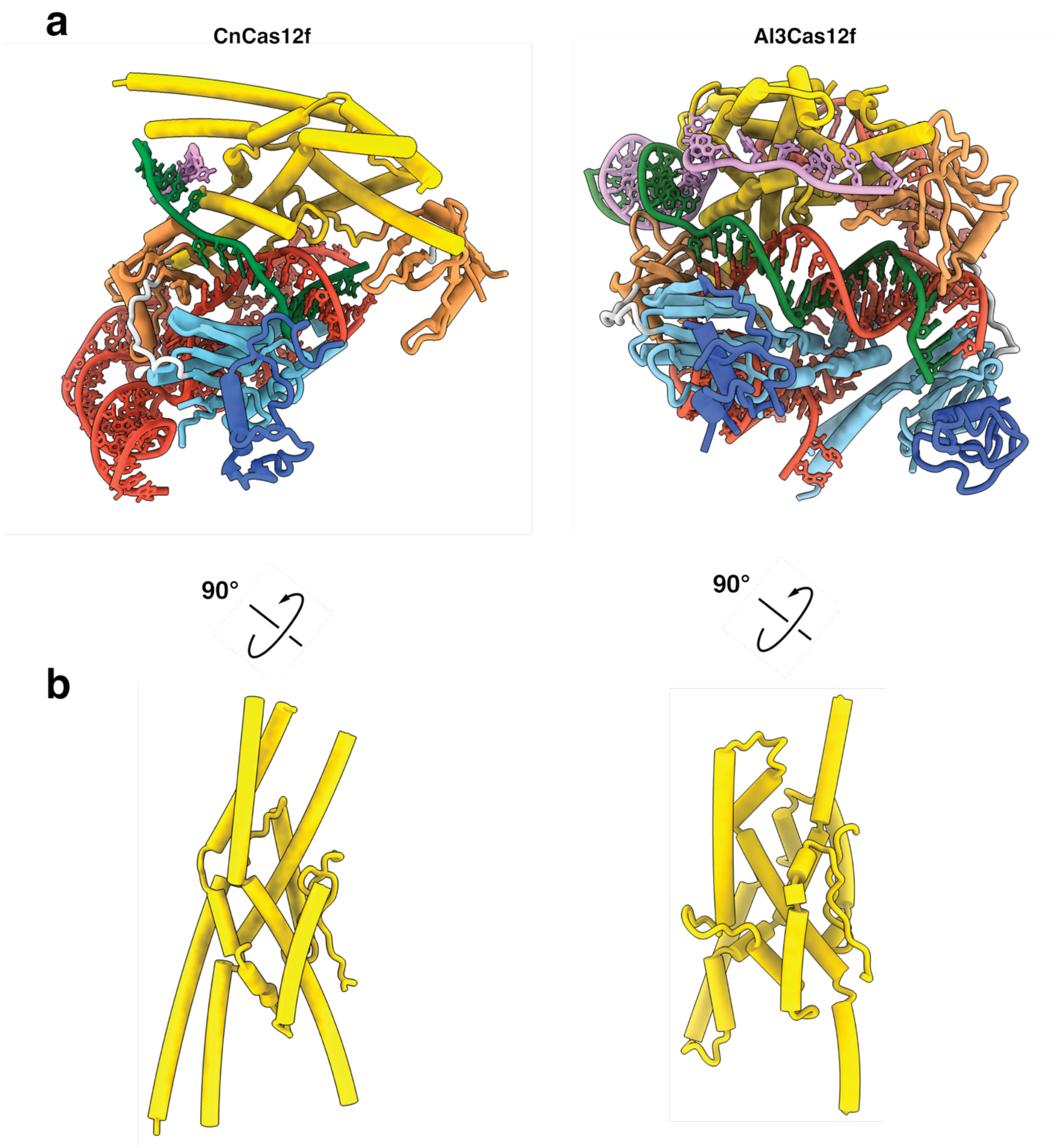

Supplementary Figure 3: Comparison between CnCas12f and Al3Cas12f.

a. Side by side comparison of the overall structures of CnCas12f (PDB: 8HR5, left) and Al3Cas12f (right). as well as **b.** the REC domains for each effector.

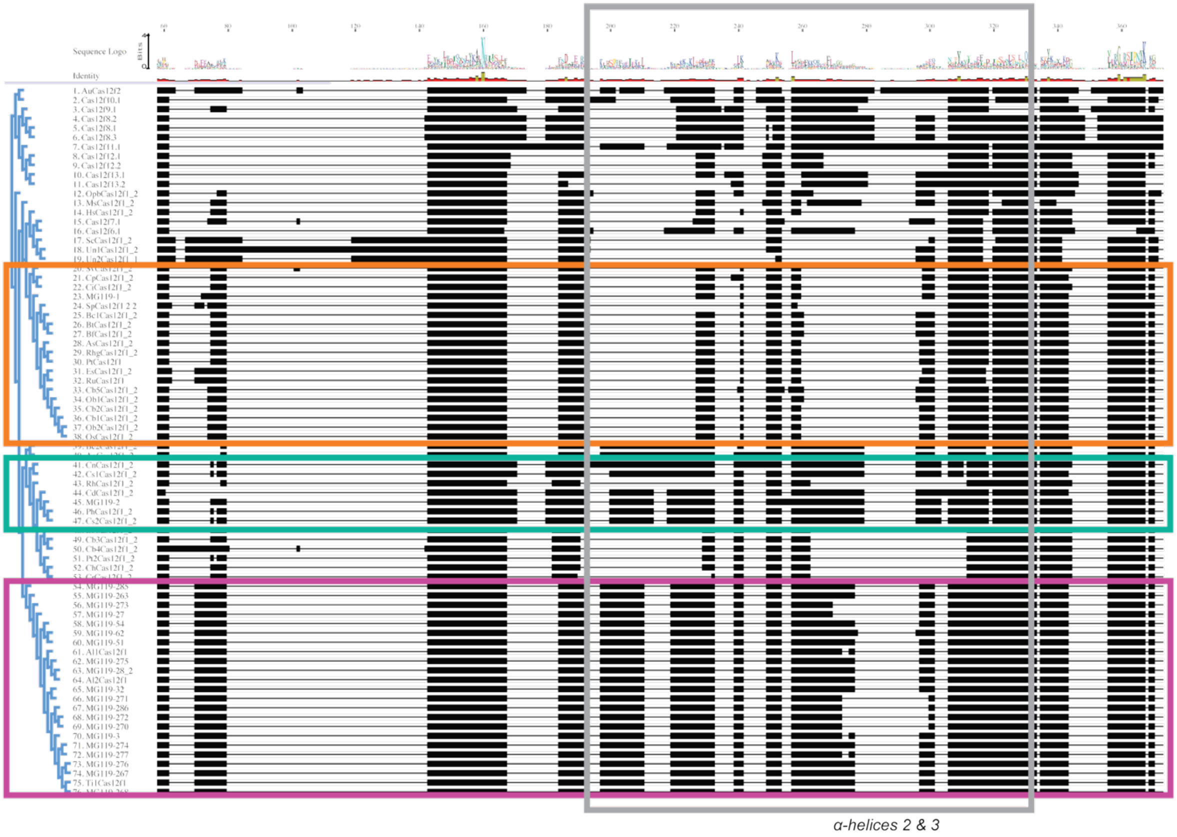

Supplementary Figure 4. Multiple sequence alignment representation of the Cas12f phylogenetic tree (FastTree).

Shown is the region comprising the REC domain. The Al3Cas12f clade is highlighted in fuchsia, a clade including CnCas12f in green, and a clade including AsCas12f1 and SpCas12f1 in orange. The region enclosed in a gray box highlights a high diversity section in the REC domain, which includes the structural alpha helices 2 and 3 in the Al3Cas12f clade (See **Suppl. Fig. 5**).

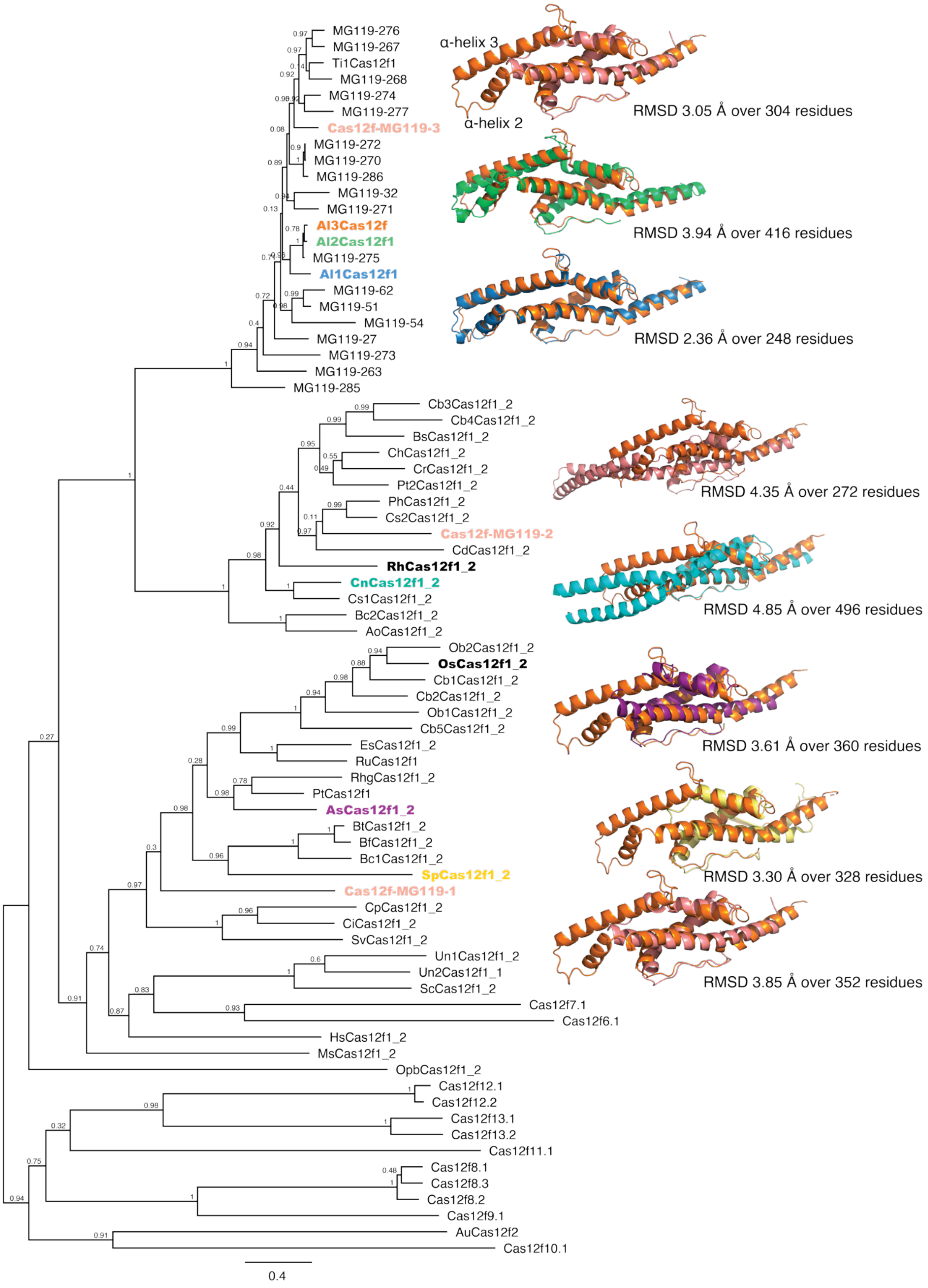

MG119-276
MG119-267
Ti1Cas12f1
MG119-268
MG119-274
MG119-277
Cas12f-MG119-3
MG119-272
MG119-270
MG119-286
MG119-32
MG119-271
Al3Cas12f
Al2Cas12f1
MG119-275
Al1Cas12f1
MG119-62
MG119-51
MG119-54
MG119-27
MG119-273
MG119-263
MG119-285
Cb3Cas12f1_2
Cb4Cas12f1_2
BsCas12f1_2
ChCas12f1_2
CrCas12f1_2
Pt2Cas12f1_2
PhCas12f1_2
Cs2Cas12f1_2
Cas12f-MG119-2
CdCas12f1_2
RhCas12f1_2
CnCas12f1_2
Cs1Cas12f1_2
Bc2Cas12f1_2
AoCas12f1_2
Ob2Cas12f1_2
OsCas12f1_2
Cb1Cas12f1_2
Cb2Cas12f1_2
Ob1Cas12f1_2
Cb5Cas12f1_2
EsCas12f1_2
RuCas12f1
RhgCas12f1_2
PtCas12f1
AsCas12f1_2
BtCas12f1_2
BfCas12f1_2
Bc1Cas12f1_2
SpCas12f1_2
Cas12f-MG119-1
CpCas12f1_2
CiCas12f1_2
SvCas12f1_2
Un1Cas12f1_2
Un2Cas12f1_1
ScCas12f1_2
Cas12f7.1
Cas12f6.1
HsCas12f1_2
MsCas12f1_2
OpbCas12f1_2
Cas12f12.1
Cas12f12.2
Cas13f13.1
Cas12f13.2
Cas12f11.1
Cas12f8.1
Cas12f8.3
Cas12f8.2
Cas12f9.1
AuCas12f2
Cas12f10.1
0.4
α-helix 3
α-helix 2
RMSD 3.05 Å over 304 residues
RMSD 3.94 Å over 416 residues
RMSD 2.36 Å over 248 residues
RMSD 4.35 Å over 272 residues
RMSD 4.85 Å over 496 residues
RMSD 3.61 Å over 360 residues
RMSD 3.30 Å over 328 residues
RMSD 3.85 Å over 352 residues

Supplementary Figure 5. Cas12f phylogenetic tree showing structural differences in the Rec domain.

Representative sequences from the literature and this work showing similarity or differences in the REC domain at the sequence level were aligned to Al3Cas12f in PyMOL using 'cealign'. Cryo-EM structures were obtained from PDB if available (8HR5, 7WJU and 9I8Y). The 3D structure of Cas12f-MG119-1, Cas12f-MG119-2, Cas12f-MG119-3, Al1Cas12f, Al2Cas12f was predicted with their corresponding gRNA using Boltz2. Given that the predicted structures are folded as monomers, shown here are only REC domains aligned to Al3Cas12f REC, even though the entire structures were aligned to each other (RMSD reported next to folds).

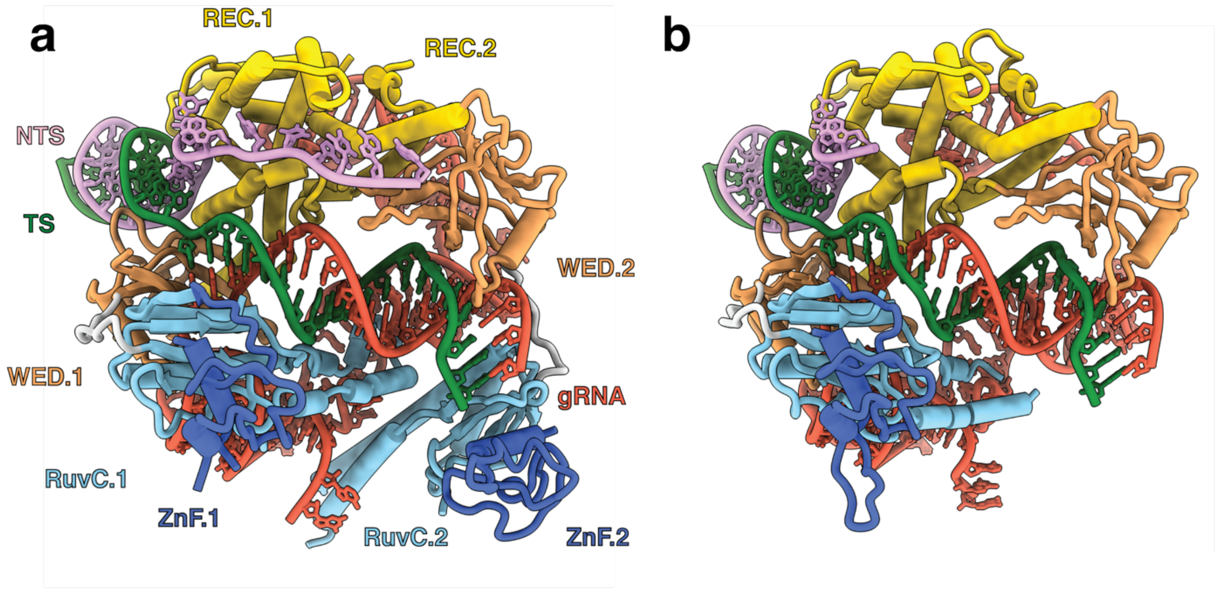

Supplementary Figure 6. Comparison of Al3Cas12f structures.

a. Al3Cas12f structure in State II. **b.** Al3Cas12f in State I. State I does not contain a resolved RuvC.2 domain. Instead, an additional unstructured region of the gRNA is resolved.

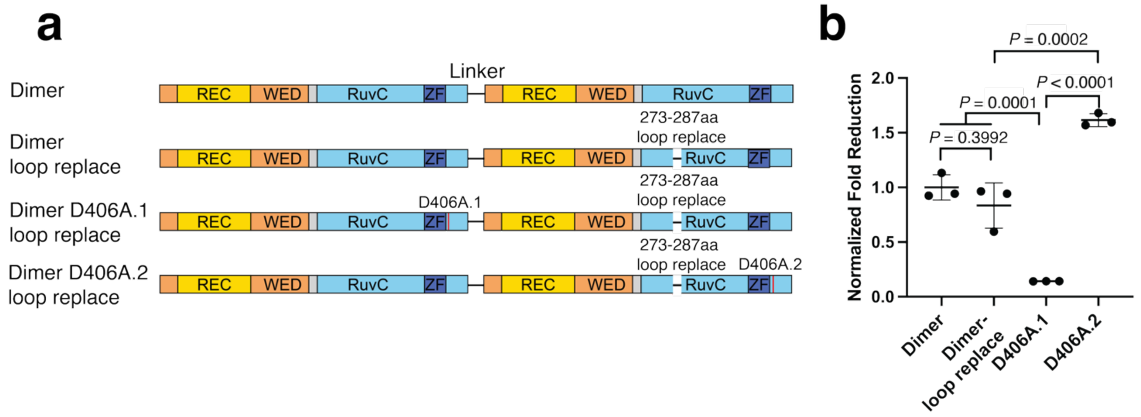

Supplementary Figure 7. Molecule 1 mediates catalytic activity in OsCas12f.

a. Design of OsCas12f covalent dimer mutants. **b.** *In vivo* activity of dimer WT and mutants of OsCas12f in GFP depletion assay. Data represent mean ± SD (n = 3 independent biological replicates). Significance determined by one-way ANOVA. Source data are provided in Source Data file.

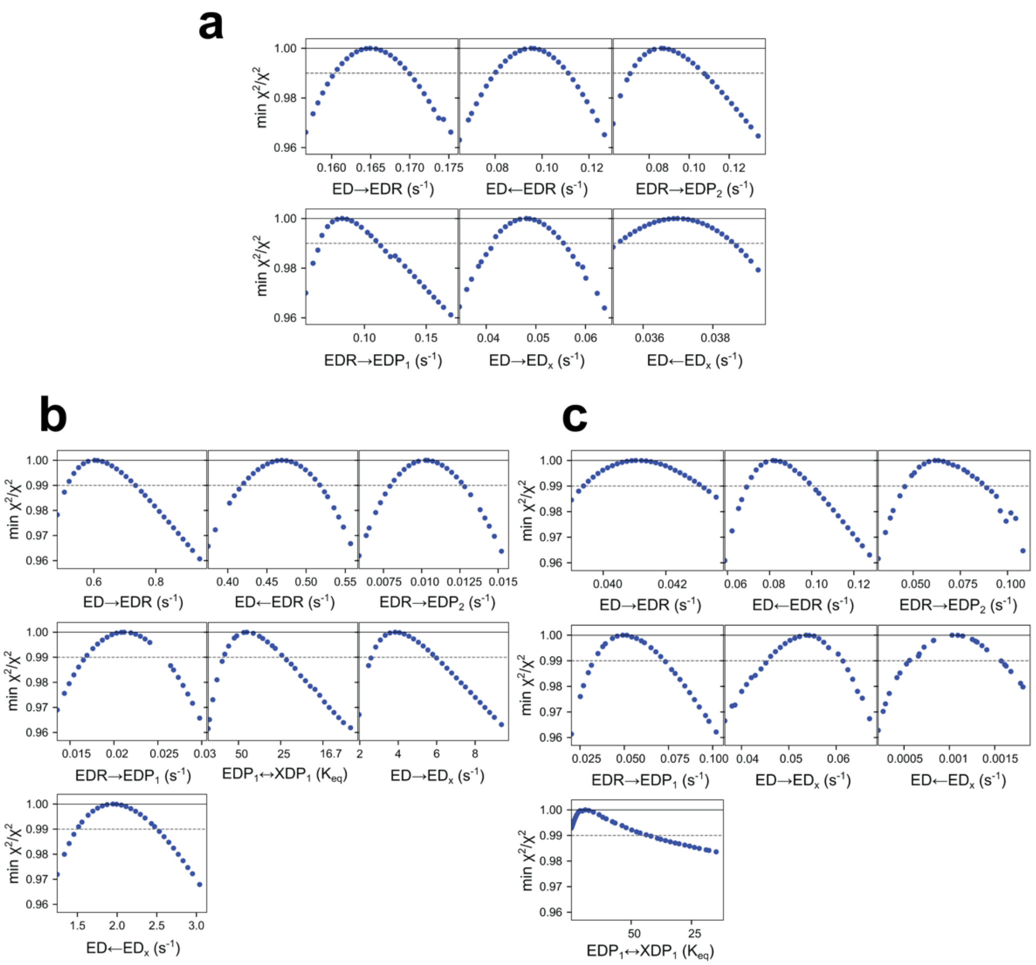

Supplementary Figure 8. Confidence contours from global data fitting for a. Al3Cas12f; b. OsCas12f; and c. RhCas12f.

Contours show the change in χ^2 as a function of values for individual rate. The dashed line indicates the χ^2 threshold corresponding to the 95% confidence interval used for reporting rates.

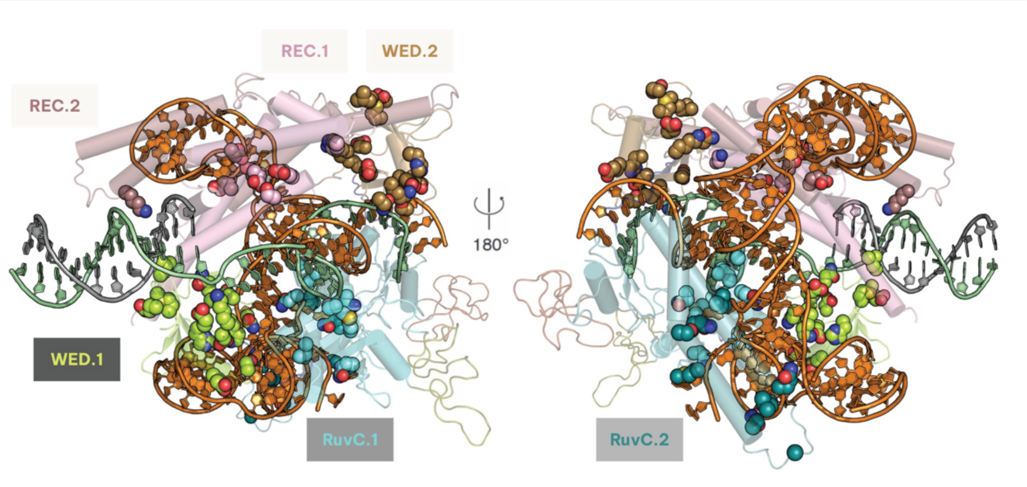

Supplementary Figure 9. Designs of point mutations for engineering of Al3Cas12f.
Locations of residues selected for single-point mutation. The residues are represented as spheres and colored by domain and atom type. DNA is represented in green (TS) and grey (NTS) while the gRNA is colored in orange.

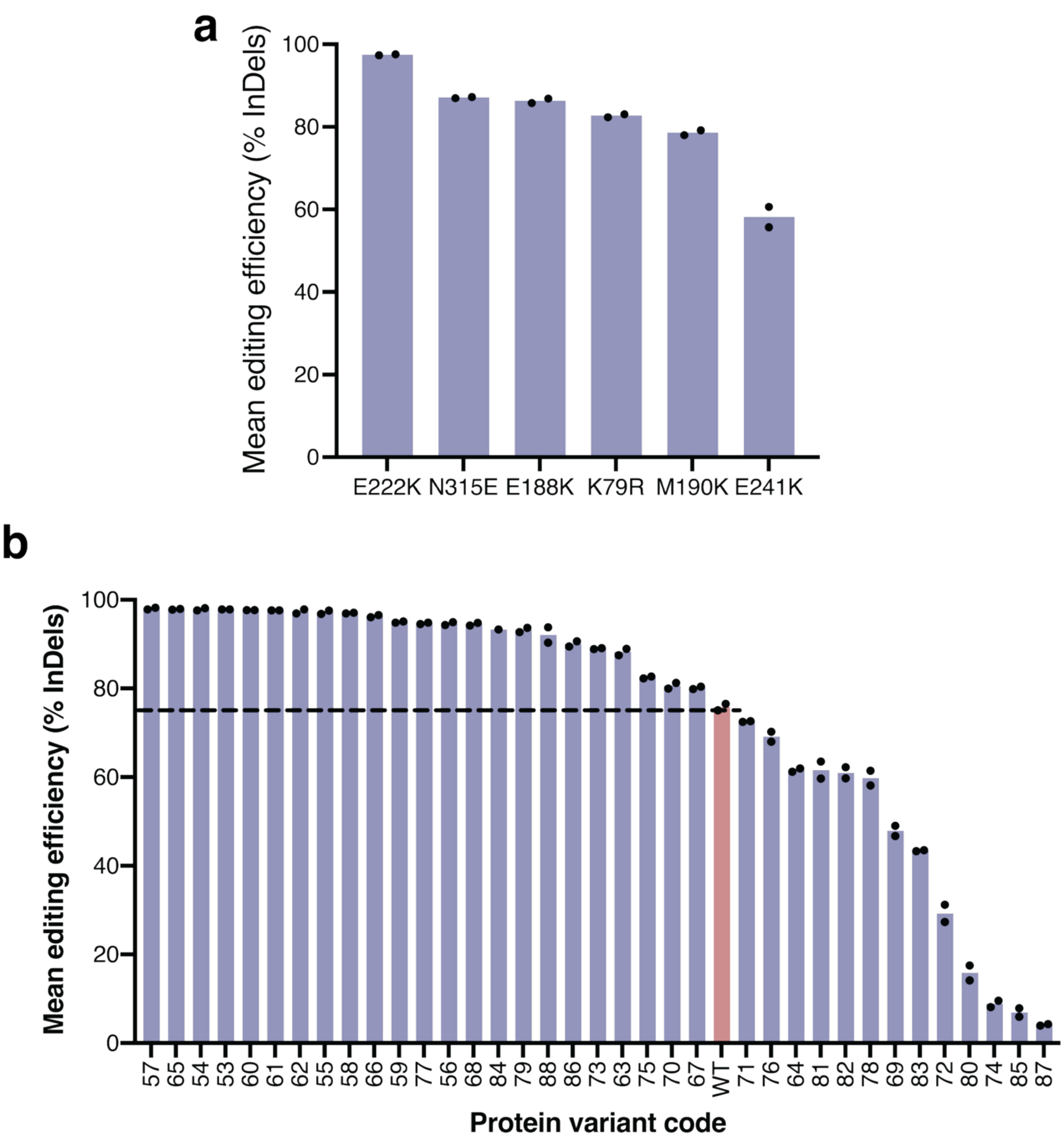

Supplementary Figure 10. Editing efficiency of single and combinatorial mutants at AAVS1 in K562 cells in comparison to WT AI3Cas12f.

a. Six single point mutation variants with editing efficiency over 60%. **b.** Editing efficiency of combinatorial mutations in comparison to WT. Bars represent the mean Indels percentage, black points represent data (n= 2 independent biological replicates, except variant # 84 with n=1) of amplicons from NGS sequencing that contain insertions or deletions obtained with each protein variant and the AAVS1 F4 spacer. Source data are provided in Source Data file.

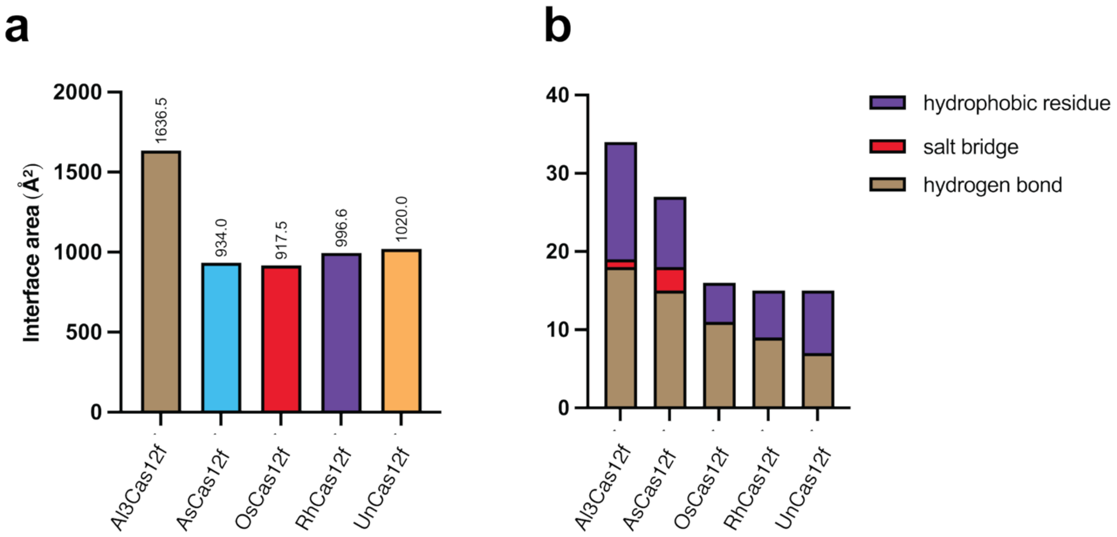

Supplementary Figure 11. Dimer interface analysis of Cas12f nucleases.

a. Interface surface area mediated by the REC domain, calculated using PDBePISA. **b.** Quantification of dimer interface interactions (hydrogen bonds, salt bridges, and hydrophobic residues). Hydrogen bonds and salt bridges were computed with PDBePISA; hydrophobic residues were manually annotated. Source data are provided in Source Data file.

Supplementary Information Source Data

Supplementary Figure 1 Source Data. Uncropped gels.

Suppl. Fig. 1a 2% agarose gel

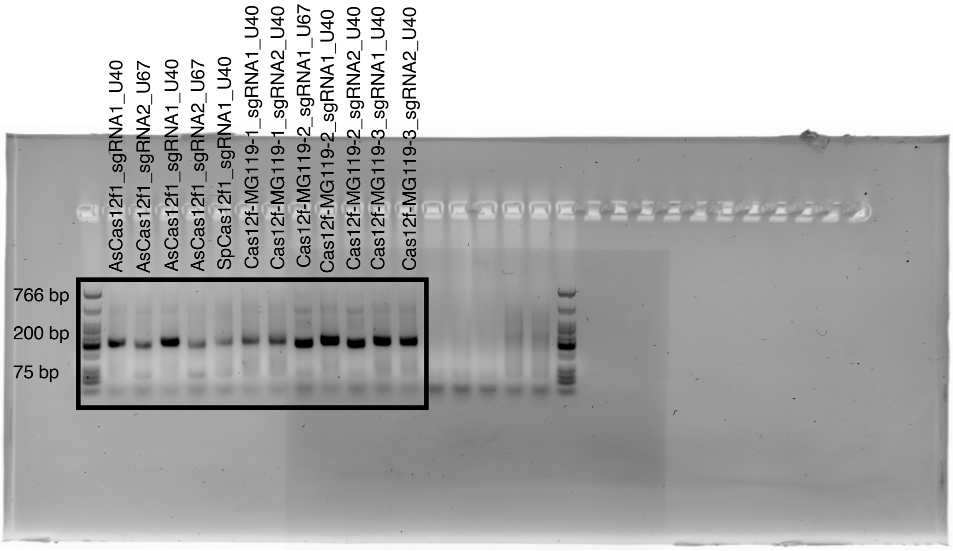

Suppl. Fig. 1b 2% agarose gel

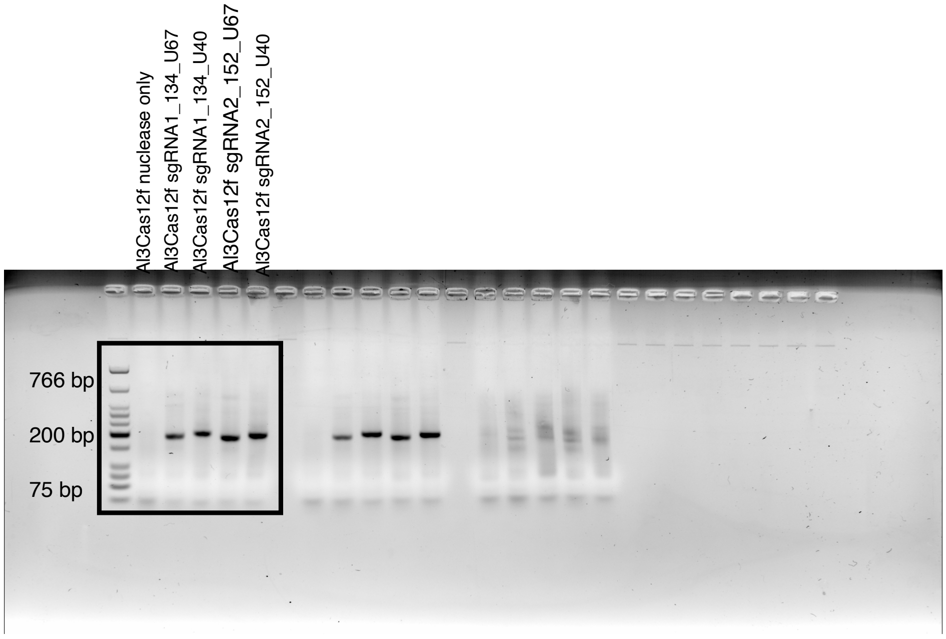

Supplementary Figure 2 Source Data. Uncropped gels.

Suppl. Fig. 2
2% agarose gel

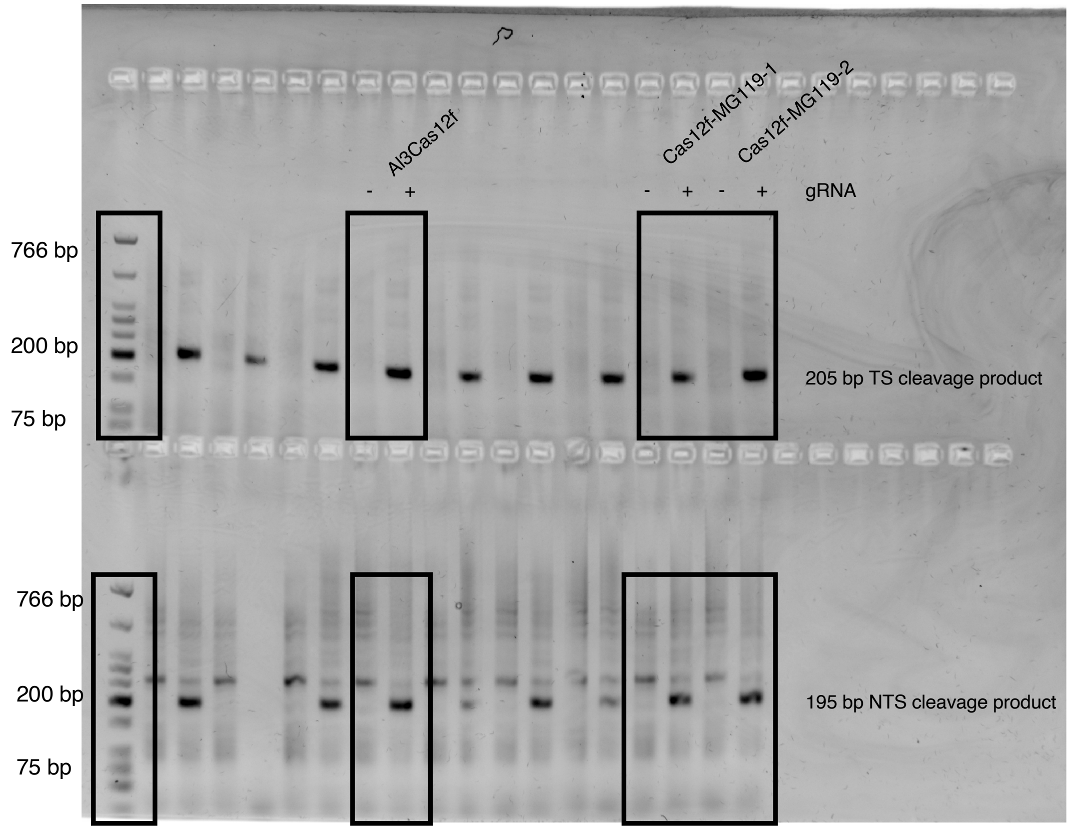

Supplement: Supplementary file 1 — Supplementary Figs. 1–11 and source data for Supplementary Figs. 1 and 2. [file 41594_2026_1788_MOESM1_ESM.pdf]
