# Supplementary material for: Comparative characterization of Cas12f orthologs reveals mechanistic features underlying enhanced genome editing efficiency
Source: Nat Struct Mol Biol. 2026 Apr 13;33(5):756–67. doi: 10.1038/s41594-026-01788-6 (PMC13186708; doi:10.1038/s41594-026-01788-6)
Supplement: Supplementary file 2 — Reporting Summary [file 41594_2026_1788_MOESM2_ESM.pdf]

nature portfolio

Corresponding author(s): David W. Taylor

Last updated by author(s): Feb 26, 2026

Reporting Summary

Nature Portfolio wishes to improve the reproducibility of the work that we publish. This form provides structure for consistency and transparency in reporting. For further information on Nature Portfolio policies, see our Editorial Policies and the Editorial Policy Checklist.

Statistics

For all statistical analyses, confirm that the following items are present in the figure legend, table legend, main text, or Methods section.

n/a	Confirmed	
☐	☒	The exact sample size (*n*) for each experimental group/condition, given as a discrete number and unit of measurement
☒	☐	A statement on whether measurements were taken from distinct samples or whether the same sample was measured repeatedly
☐	☒	The statistical test(s) used AND whether they are one- or two-sided<br>*Only common tests should be described solely by name; describe more complex techniques in the Methods section.*
☒	☐	A description of all covariates tested
☒	☐	A description of any assumptions or corrections, such as tests of normality and adjustment for multiple comparisons
☐	☒	A full description of the statistical parameters including central tendency (e.g. means) or other basic estimates (e.g. regression coefficient) AND variation (e.g. standard deviation) or associated estimates of uncertainty (e.g. confidence intervals)
☐	☒	For null hypothesis testing, the test statistic (e.g. *F*, *t*, *r*) with confidence intervals, effect sizes, degrees of freedom and *P* value noted<br>*Give P values as exact values whenever suitable.*
☒	☐	For Bayesian analysis, information on the choice of priors and Markov chain Monte Carlo settings
☒	☐	For hierarchical and complex designs, identification of the appropriate level for tests and full reporting of outcomes
☒	☐	Estimates of effect sizes (e.g. Cohen's *d*, Pearson's *r*), indicating how they were calculated

Our web collection on statistics for biologists contains articles on many of the points above.

Software and code

Policy information about availability of computer code

Data collection	Grids were screened with SerialEM on a FEI Glacios cryo-TEM. For Cas12f-MG119-28 and OsCas12f ternary complexes, images were collected on a FEI Glacios cryo-TEM equipped with a Falcon 4 detector with a pixel size of 0.933 Å, while the images of the RhCas12f ternary complex were collected on a FEI Titan Krios cryo-TEM equipped with a Gatan K3 direct electron detector with a pixel size of 0.8332 Å. The defocus range was set to -1.5 to -2.5 µm. Motion correction, contrast transfer function (CTF) estimation and particle picking were carried out in cryoSPARC live v4.0.
Data analysis	All subsequent data processing was carried out in cryoSPARC v4.4. The Cas12f protein structure predicted by AlphaFold2 was fitted into the ternary complex map as a rigid body in ChimeraX. gRNA was modeled based on secondary structure prediction and gRNA architecture of AsCas12f. The model was manually refined in COOT 43, and automatically refined by real_space_refine in PHENIX. Cas12f-MG119-28 was built using Cosmic2 ModelAngelo. The model was subsequently manually refined using COOT and Isolde, and automatically refined by real_space_refine in PHENIX. All structural figures were generated using ChimeraX v1.7.1.

For manuscripts utilizing custom algorithms or software that are central to the research but not yet described in published literature, software must be made available to editors and reviewers. We strongly encourage code deposition in a community repository (e.g. GitHub). See the Nature Portfolio guidelines for submitting code & software for further information.

Data

Policy information about availability of data

All manuscripts must include a data availability statement. This statement should provide the following information, where applicable:

- Accession codes, unique identifiers, or web links for publicly available datasets
- A description of any restrictions on data availability
- For clinical datasets or third party data, please ensure that the statement adheres to our policy

Structures of the Cas12f-MG119-28 State I, State II, OsCas12f State I, State II, State III, RhCas12f have been deposited in the EMDB with accession codes: EMD-49954, EMD-49957, EMD-49959, EMD-49956, EMD-49958, EMD-49955, respectively. Associated atomic coordinates were deposited to PDB with accession codes: 9NZO, 9NZR, 9NZT, 9NZQ, 9NZS, 9NZP, respectively. Protein and guide RNA sequences for nucleases reported here are available in Supplemental Data tables.

Research involving human participants, their data, or biological material

Policy information about studies with human participants or human data. See also policy information about sex, gender (identity/presentation), and sexual orientation and race, ethnicity and racism.

Reporting on sex and gender	Not applicable.
Reporting on race, ethnicity, or other socially relevant groupings	Not applicable.
Population characteristics	Not applicable.
Recruitment	Not applicable.
Ethics oversight	Not applicable.

Note that full information on the approval of the study protocol must also be provided in the manuscript.

Field-specific reporting

Please select the one below that is the best fit for your research. If you are not sure, read the appropriate sections before making your selection.

☒ Life sciences ☐ Behavioural & social sciences ☐ Ecological, evolutionary & environmental sciences

For a reference copy of the document with all sections, see nature.com/documents/nr-reporting-summary-flat.pdf

Life sciences study design

All studies must disclose on these points even when the disclosure is negative.

Sample size	A sample size of three independent biological replicates were required to derive statistical measurements. A sample size of two was chosen for initial screening purposes to show a general trend.
Data exclusions	No data was excluded.
Replication	All experiments were replicated at least three times with similar results.
Randomization	Particle orientations are randomized during processing.
Blinding	No blinding was performed. The experiments were observational and not subject to bias.

Reporting for specific materials, systems and methods

We require information from authors about some types of materials, experimental systems and methods used in many studies. Here, indicate whether each material, system or method listed is relevant to your study. If you are not sure if a list item applies to your research, read the appropriate section before selecting a response.

Materials & experimental systems

n/a	Involved in the study	
☒	☐	Antibodies
☐	☒	Eukaryotic cell lines
☒	☐	Palaeontology and archaeology
☒	☐	Animals and other organisms
☒	☐	Clinical data
☒	☐	Dual use research of concern
☒	☐	Plants

Methods

n/a	Involved in the study	
☒	☐	ChIP-seq
☒	☐	Flow cytometry
☒	☐	MRI-based neuroimaging

Eukaryotic cell lines

Policy information about cell lines and Sex and Gender in Research

Cell line source(s)	K562 cells were used in cell editing experiments.
Authentication	Cells were purchased from ATTC and cultured using manufacturer protocols.
Mycoplasma contamination	Cell lines were not tested for Mycoplasma contamination.
Commonly misidentified lines (See ICLAC register)	Non used.

Plants

Seed stocks	Not applicable.
Novel plant genotypes	Not applicable.
Authentication	Not applicable.
